# Supplementary material for: Chemical characterization, antimicrobial, and antioxidant potentials of Juniperus phoenicea wood tar
Source: PLoS One. 2026 Apr 1;21(4):e0345923. doi: 10.1371/journal.pone.0345923 (PMC13043053; doi:10.1371/journal.pone.0345923)
Supplement: Attachment — Submitted filename: plos.docx [file pone.0345923.s001.docx]

Manuscript: “Chemical characterization, antimicrobial, and antioxidant potentials of Juniperus phoeniceae wood tar”.

The manuscript provides general information on the antibacterial and antioxidant activities of Juniperus phoenicea wood tar (JPWT), supported by qualitative and quantitative phytochemical analyses. However, it requires thorough English language revision and addressing of the following major issues:

**Title**

Correct "Juniperus phoeniceae" to “Juniperus phoenicea”. Please go throughout the entire manuscript.

**Abstract**

Correct "Juniperus phoeniceae" to “Juniperus phoenicea L.” in the first citation.

Rewrite "confirming its free radical-scavenging capability" to "confirming its antioxidant activity." Note that "radical-scavenging capability" is appropriate only when using radicals such as DPPH.

**Materials and Methods**

Rewrite the sentence "Voucher specimen of JP is deposited in the Herbarium (RAB114890) of the Scientific Institute of Rabat (Morocco) (Branches were examined for integrity and absence of dust and insect contamination)" for clarity and completeness.

Rename section "2.3. Preliminary phytochemical tests" to "2.3. Qualitative phytochemical tests."

Section 2.3.1: Avoid starting sentences with numbers.

Rewrite "After adding 2 mL of distilled water was added to 2 mL ethanolic solution of JPWT" for grammatical accuracy.

Correct "2.4.2. Flavonoid" to "2.4.2. Flavonoids."

Correct "2.4.3. Tannin" to "2.4.3. Tannins."

Correct "2.5.1. 2,2-Diphenyl-1-picrylhydrazylradical assay (DPPH)" to "2.5.1. 2,2-Diphenyl-1-picrylhydrazyl (DPPH) radical assay."

Correct "Tests were carried out in triplicate using ascorbic acid and BHT has positive control" to "Tests were carried out in triplicate using ascorbic acid and BHT as positive controls."

Use consistent nomenclature throughout (e.g., J. phoeniceae or JPWT instead of "wood cedar" in section 2.5.1).

Specify the tested microorganisms in the sentence: "A suspension of the tested microorganism (0.5 mL of 106 cells/mL) was spread on nutrient agar."

Define abbreviations in the sentence: "Positive controls were prepared using the CN and IPM antibiotics."

Section 2.7. GC-MS analysis: Clarify how phytocompounds were identified.

Consider switching to HPLC-MS, which is more suitable for polyphenol, alkaloid, and flavonoid-rich extracts.

Define BSTFA.

**Results**

Correct all "Tableau X" to "Table X" (e.g., Table 1, Table 2… Table 6).

Use decimal points (.) consistently in values for Tables 2 and 3.

In Table 3, specify units for IC50

Section 3.4. GC-MS analysis: Avoid detailing retention times (RT) of identified compounds.

Correct "3.5. Antibacterial stains" to "3.5. Antibacterial activity."

Please insert the sentence "The antibacterial activity of JPWT was tested against both Gram-positive and Gram-negative bacteria at concentrations of 25 mg/mL and 50 mg/mL" in the Materials and Methods section.

Italicize scientific names (e.g., Staphylococcus aureus).

**Discussion**

Remove "Firstly,".

Rewrite "Phytochemical screening revealed the presence of alkaloids, tannins, phenols, and flavonoids (Table 1). Quantification of total phenolics, tannins, and flavonoids (Table 2) by UV-Visible spectroscopy demonstrated that JPWT is rich in these compound", to include in-depth literature-based discussion specific to J. phoeniceae.

Rewrite Table 5 title to "Inhibition zone diameter (mm) of Juniperus phoenicea wood tar and positive controls (antibiotics) against selected bacteria."

Report values in Table 5 as means of three replicates ± standard deviation, with appropriate statistical analysis.

**Conclusions**

Correct "Collectively, these results position JPWT as a valuable reservoir of bioactive natural products with considerable pharmaceutical and therapeutic promise" for grammatical accuracy and precision.

**References**

Complete the reference : El Jemli M. "Contribution to the ethnobotanical, toxicological, pharmacological and phytochemical study of four Moroccan Cupressaceae: Juniperus thurifera L., Juniperus oxycedrus L., Juniperus phoenicea L. and Tetraclinis articulata L." (Provide full details: thesis/degree, institution, year, etc., 2020).

Please recheck the whole reference list.
