# Supplementary material for: Chemical characterization, antimicrobial, and antioxidant potentials of Juniperus phoenicea wood tar
Source: PLoS One. 2026 Apr 1;21(4):e0345923. doi: 10.1371/journal.pone.0345923 (PMC13043053; doi:10.1371/journal.pone.0345923)
Supplement: Attachment — Submitted filename: Commentaires des reviewers de plos one 01.docx [file pone.0345923.s003.docx]

**The authors sincerely thank the editor and the reviewers for their valuable time, consideration, and suggestions. The constructive comments have significantly enhanced the overall quality of the manuscript. We trust that we have adequately addressed your inquiries and concerns. All molecular docking data generated in this study, including docking poses, 2D/3D interaction diagrams, binding energies, and related results, have been made available through a free and open-access web repository to ensure transparency and reproducibility. These data can be accessed at:**[**https://github.com/khibech/Juniperus-phoenicea-**](https://github.com/khibech/Juniperus-phoenicea-)**. In addition, the Funding section has been updated in the revised manuscript, and the funding year has been corrected to 2026 in order to avoid any misunderstanding and to ensure the accuracy of the funding information.**

**Manuscript Number: PLONE-D-25-64294**

Paper Title: Chemical characterization, antimicrobial, and antioxidant potentials of *Juniperus phoenicea* wood tar

**Reviewer #1:**

Manuscript: “Chemical characterization, antimicrobial, and antioxidant potentials of Juniperus phoeniceae wood tar”.
The manuscript provides general information on the antibacterial and antioxidant activities of Juniperus phoenicea wood tar (JPWT), supported by qualitative and quantitative phytochemical analyses. However, it requires thorough English language revision and addressing of the following major issues:

Title

1. Correct "Juniperus phoeniceae" to “Juniperus phoenicea”. Please go throughout the entire manuscript.

Thank you for your remark, we corrected the name of the specie to “Juniperus phoeniceae” to Juniperus Phoenicea in the title and the entire manuscript.

Abstract

2. Correct "Juniperus phoeniceae" to “Juniperus phoenicea L.” in the first citation.

Thank you for this valuable remark, we corrected "Juniperus phoeniceae" to “Juniperus phoenicea L.” in the first citation (Abstract section, introduction and 2.1. Plant materials)

3. Rewrite "confirming its free radical-scavenging capability" to "confirming its antioxidant activity." Note that "radical-scavenging capability" is appropriate only when using radicals such as DPPH

Thank you for this suggestion. We rewrote "confirming its free radical-scavenging capability" to "confirming its antioxidant activity" in the abstract

Materials and Methods

1. Rewrite the sentence "Voucher specimen of JP is deposited in the Herbarium (RAB114890) of the Scientific Institute of Rabat (Morocco) (Branches were examined for integrity and absence of dust and insect contamination)" for clarity and completeness.

Thank you for this comment, Section 2.1.” Plant materials” was rewritten for clarity and completeness, and we added the declaration of authority permits.

2. Rename section "2.3. Preliminary phytochemical tests" to "2.3. Qualitative phytochemical tests."
   Section 2.3.1: Avoid starting sentences with numbers.

Thank you for your suggestion. Section "2.3. Preliminary phytochemical tests" was renamed to "2.3. Qualitative phytochemical tests." Section 2.3.1. has been revised to avoid starting with a number and correcting the numeration of Section 2.3.6 to

3. Rewrite "After adding 2 mL of distilled water was added to 2 mL ethanolic solution of JPWT" for grammatical accuracy.

Thank you for this remark, we corrected the grammatical accuracy and rewrite the section to be more fluent.

4. Correct "2.4.2. Flavonoid" to "2.4.2. Flavonoids."

Thank you for your suggestion, we corrected Flavonoid" to "2.4.2. Flavonoids

5. Correct "2.4.3. Tannin" to "2.4.3. Tannins."

Thank you for your suggestion, we corrected "2.4.3. Tannin" to "2.4.3. Tannins."

6. Correct "2.5.1. 2,2-Diphenyl-1-picrylhydrazylradical assay (DPPH)" to "2.5.1. 2,2-Diphenyl-1-picrylhydrazyl (DPPH) radical assay."

Thank you for remark, we corrected "2.5.1. 2,2-Diphenyl-1-picrylhydrazylradical assay (DPPH)" to "2.5.1. 2,2-Diphenyl-1-picrylhydrazyl (DPPH) radical assay."

7. Correct "Tests were carried out in triplicate using ascorbic acid and BHT has positive control" to "Tests were carried out in triplicate using ascorbic acid and BHT as positive controls."
   Use consistent nomenclature throughout (e.g., J. phoeniceae or JPWT instead of "wood cedar" in section 2.5.1).

Thank you for these suggestions, we replaced Tests were carried out in triplicate using ascorbic acid and BHT has positive control" to "Tests were carried out in triplicate using ascorbic acid and BHT as positive controls." We are using consistent nomenclature, corrected 50µl/mL by 50µL and clarify the description.

8. Specify the tested microorganisms in the sentence: "A suspension of the tested microorganism (0.5 mL of 106 cells/mL) was spread on nutrient agar."

Thank you for this comment, we specify all microorganism tested in Section “2.6 Disc Diffusion assay”

9. Define abbreviations in the sentence: "Positive controls were prepared using the CN and IPM antibiotics."

Thank you for this comment, we defined the positive control CN and IPM antibiotics

10. Section 2.7. GC-MS analysis: Clarify how phytocompounds were identified.

Consider switching to HPLC-MS, which is more suitable for polyphenol, alkaloid, and flavonoid-rich extracts.
Define BSTFA.

Thank you for your suggestion, we defined BSTFA and added identification phrases to clarify how phytocompounds were identified

Results

1. Correct all "Tableau X" to "Table X" (e.g., Table 1, Table 2… Table 6).
2. Use decimal points (.) consistently in values for Tables 2 and 3.
3. In Table 3, specify units for IC50

Thank you for all these comments. The table legends have been corrected "Tableau X" to "Table X" (e.g., Table 1, Table 2… Table 6), the decimal formatting has been standardized, and Table 3 now specifies IC₅₀ values in µg/mL.

4. Section 3.4. GC-MS analysis: Avoid detailing retention times (RT) of identified compounds.
   Correct "3.5. Antibacterial stains" to "3.5. Antibacterial activity."

Thank you for this valuable remark, Section 3.4 has been rewritten to focus on the major compounds and their relative percentages, without listing all retention times. We changed "3.5. Antibacterial stains" to "3.5. Antibacterial activity."

5. Please insert the sentence "The antibacterial activity of JPWT was tested against both Gram-positive and Gram-negative bacteria at concentrations of 25 mg/mL and 50 mg/mL" in the Materials and Methods section.

Thank you, we insert the sentence "The antibacterial activity of JPWT was tested against both Gram-positive and Gram-negative bacteria at concentrations of 25 mg/mL and 50 mg/mL" in the Materials and Methods section

6. Italicize scientific names (e.g., Staphylococcus aureus).

Thank you, the Italicize the scientific name of (e.g.,Staphylococcus aureus)

Discussion

1. Remove "Firstly,". Rewrite "Phytochemical screening revealed the presence of alkaloids, tannins, phenols, and flavonoids (Table 1). Quantification of total phenolics, tannins, and flavonoids (Table 2) by UV-Visible spectroscopy demonstrated that JPWT is rich in these compound", to include in-depth literature-based discussion specific to J. phoeniceae.

Thank you for this suggestion. We have removed “Firsty.” The discussion has been rewritten, clarified, and strengthened with recent references on *Juniperus phoenicea*, as well as an ethnobotanical reference on tar production and use (refs. [59-61]).

2. Rewrite Table 5 title to "Inhibition zone diameter (mm) of Juniperus phoenicea wood tar and positive controls (antibiotics) against selected bacteria."

Thank you for this suggestion, we rewrite Table 5 title to "Inhibition zone diameter (mm) of Juniperus phoenicea wood tar and positive controls (antibiotics) against selected bacteria."

3. Report values in Table 5 as means of three replicates ± standard deviation, with appropriate statistical analysis.

Thank you for your comment. We report values in Table 5 as means of three replicates ± standard deviation, with appropriate statistical analysis in all tables.

Conclusions

1. Correct "Collectively, these results position JPWT as a valuable reservoir of bioactive natural products with considerable pharmaceutical and therapeutic promise" for grammatical accuracy and precision.

Thank you for this remark, Section conclusion was corrected and rewrite by adding limit, valorization and perspectives.

References

2. Complete the reference: El Jemli M. "Contribution to the ethnobotanical, toxicological, pharmacological and phytochemical study of four Moroccan Cupressaceae: Juniperus thurifera L., Juniperus oxycedrus L., Juniperus phoenicea L. and Tetraclinis articulata L." (Provide full details: thesis/degree, institution, year, etc., 2020).
   Please recheck the whole reference list.

Thank you for your suggestion, we completed the reference (ref [25]) and rechek the whole reference list.

**Reviewer #2:**

Submission ID: PONE-D-25-64294
Journal: PLOS ONE
Dear authors,
Your manuscript entitled “Chemical Characterization, antimicrobial, and antioxidant potentials of Juniperus phoeniceae wood tar” has been reviewed. The topic of your manuscript is very interesting, you are following the right scientific path for writing your manuscript, and the manuscript is well organised and presents important information for the scientific field. However, the manuscript needs some improvement. Therefore, I would like to make some comments, recommendations about the revised manuscript:

•The objective of your research should be discussed in detail in the last paragraph of the introduction.
Thank you for this valuable comment, we will explain the main objective of our research carefully at the end of the Introduction Section.

• What is the importance of adding a Molecular Docking section? Normally, the in-silico study should be carried out before starting the study.

The Molecular Docking section is important because it provides a structural and mechanistic understanding of ligand–protein interactions, including binding orientation and key stabilizing contacts. Although in silico studies are ideally conducted prior to initiating a study, the inclusion of molecular docking in the present work allows us to rationalize the binding mechanism and to strengthen the interpretation of the computational results within a coherent in silico workflow.

• What is the added value of Molecular Docking for your study?
The added value of Molecular Docking in our study lies in its ability to validate the biologically relevant binding site, identify the key ligand–protein interactions, and generate reliable initial binding poses.

• The references cited in the Molecular Docking section are not compatible with the contents. Please check these references.
Thank you for this comment. We confirm that the references related to the Molecular Docking section have been carefully checked and verified.

• Add a section for statistical analysis and add the results of the statistical analysis.

Thank you for this remark, we added a new section in Materials and Methods by detailing the statistical analysis. All tables were adjusted by adding statistical output.

• The Discussion section requires a recent reference in the relevant fields and should show the socio-economic importance of your results in relation to those in the literature.

Thank you for this comment, the Discussion has been enriched with recent references and an expanded discussion on socio-economic/ethnobotanical importance and sustainability (refs. [30,49,59-61]).

• For the conclusion, present the most important results, the socio-economic importance of the results obtained, as well as recommendations and prospects for your research.

The Conclusion has been reformulated and now includes the main findings, study limitations (experimental validation), and future perspectives (compound isolation, MIC/MBC determination, in vivo studies, additional LC–MS/MS analyses, and sustainable valorization).
