# Supplementary material for: Chemical characterization, antimicrobial, and antioxidant potentials of Juniperus phoenicea wood tar
Source: PLoS One. 2026 Apr 1;21(4):e0345923. doi: 10.1371/journal.pone.0345923 (PMC13043053; doi:10.1371/journal.pone.0345923)
Supplement: Attachment — Submitted filename: Plos one aswers revision 2.docx [file pone.0345923.s004.docx]

**The authors sincerely thank the editor and the reviewers for their valuable time, consideration, and suggestions. The constructive comments have significantly enhanced the overall quality of the manuscript. We trust that we have adequately addressed your inquiries and concerns.**

**Manuscript Number: PONE-D-25-64294R1**

**Paper Title:** Chemical characterization, antimicrobial, and antioxidant potentials of Juniperus phoenicea wood tar.

**Reviewer 1:**

The authors have addressed most of the previous comments, but some points require further revision and clarification. Thorough English language revision is still needed throughout the manuscript.

Thank you for this remarkable remark. We will revise the document by correcting grammar and correcting certain English language errors.

Reference(s) is needed for this statement: "Among its derivatives, Juniperus phoeniceae wood tar (JPWT), obtained by the destructive distillation of JP wood, is traditionally used for skin ailments and infections."

Thank you for this comment, the reference for this part was added (ref 14).

Correct "Juniperus phoeniceae” in the whole document.

Thank you for this remark, we correct “juniperus phoniceae” to “juniperus phonicea” in the whole document.

Revise references for qualitative phytochemical tests, ensuring the use of relevant references and correct numbering/formatting according to journal instructions.

Thank you for your comment, we revise the reference and formatting according to journal's instructions.

Specify the amount of each antibiotic/disc.

Thank you for this comment. We have added the amount of each antibiotic/disc.

State exactly the number of replicates in the Statistical analysis section.
Avoid starting sentences with numbers.

Thank you, we added the number (n=3) of replicates in the statistical analysis section.

Provide the source/references for the MRSA, ESBL-EC, and CREC strains used in this study, and complete the references for ATCC strains as well.

Thank you, we have added the source of the bacteria “The following bacterial strains were obtained at the Research Technology Platforms of Mohamed VI University of Sciences and Health” and the reference for each bacterium in section 2.6. Disc Diffusion Assay.

Revise the chemical composition table, as the percentage areas sum to more than 100%. Clarify how these percentages were calculated and how identification was performed. Add the chromatogram if applicable.

Thank you for this comment. The initial data provided by the technician were expressed in counts rather than peak areas. After contacting the technician, we obtained the raw chromatographic data, from which the peak areas were automatically calculated by the instrument’s software. The revised table now includes the following parameters: Peak, name of compounds, RT and Area. We added the chromatogram (Fig. 1).

Please include the statistical significance in the tables 3 & 5, rather than in the footnote for greater clarity.

Thank you for this comment, we added the statistical report to the all table. Different superscript letters within the same row indicate significant differences according to Tukey’s test.

Revise the sequence and order of headings (eg. chemical composition, antioxidant, antibacterial sections) to ensure logical flow.

Thank you for your suggestion, we reorganize the sequence order to ensure a logical flow. We reported respectively preliminary phytochemical tests, GC-MS report- Antioxidant activities-antibacterial activity and finished with Molecular docking.

**Reviewer #2:**

Dear authors,
With these significant revisions, I consider the article suitable for publication.

We sincerely thank the reviewer for the careful evaluation of our manuscript and for considering it suitable for publication after the revisions. We greatly appreciate the constructive comments and suggestions, which have helped us improve the quality and clarity of the manuscript.
